# Metabolite Profiling of “Green” Extracts of *Cynara cardunculus* subsp. *scolymus*, Cultivar “Carciofo di Paestum” PGI by ^1^H NMR and HRMS-Based Metabolomics

**DOI:** 10.3390/molecules27103328

**Published:** 2022-05-22

**Authors:** Antonietta Cerulli, Milena Masullo, Cosimo Pizza, Sonia Piacente

**Affiliations:** Dipartimento di Farmacia, Università degli Studi di Salerno, Via Giovanni Paolo II n. 132, 84084 Fisciano, SA, Italy; acerulli@unisa.it (A.C.); mmasullo@unisa.it (M.M.); pizza@unisa.it (C.P.)

**Keywords:** *Cynara cardunculus* subsp. *scolymus* “Carciofo di Paestum” PGI, green extracts, ^1^H NMR and HRMS-based metabolomics, multivariate data analysis

## Abstract

Globe artichoke (*Cynara cardunculus* L. var. *scolymus* L.), is a perennial plant widely cultivated in the Mediterranean area, known for its edible part named capitula or heads. Its functional properties are related to its high levels of polyphenolic compounds and inulin. “Carciofo di Paestum”, an Italian traditional cultivar, is a labeled PGI (Protected Geographical Indication) product of the Campania region, representing an important economic resource. So far, a few chemical investigations were performed on this cultivar, mainly focused on the analysis of methanol extracts. Due to the increasing use of food supplements, in this study, a comprehensive analysis of green extracts of “Carciofo di Paestum” PGI heads was performed. EtOH, EtOH: H_2_O (80:20, 70:30, 60:40) extracts, as well as infusions and decoctions prepared according to Pharmacopeia XII were analyzed by LC-ESI/QExactive/MS/MS. A total of 17 compounds corresponding to caffeoylquinic acid derivatives, phenolics, flavonoids, and terpenoids were identified. The extracts were further submitted to NMR analysis to highlight the occurrence of primary metabolites. Both LCMS and NMR data were analyzed by Principal Component Analysis (PCA), showing significant differences among the extraction methods. Moreover, 5-caffeoylquinic acid and 1,5-dicaffeoylquinic acid were quantified in the extracts by LC-ESI/QTrap/MS/MS using the Multiple Reaction Monitoring (MRM) method. Furthermore, the phenolic content, antioxidant activity, and α-glucosidase inhibitory activity of *C. cardunculus* var. *scolymus* “Carciofo di Paestum” extracts were evaluated.

## 1. Introduction

Globe artichoke (*Cynara cardunculus* L. var. *scolymus* L.), belonging to the family Asteraceae, is a perennial plant widely cultivated in the Mediterranean area. The edible parts of the plant are the large immature inflorescences, named capitula or heads, with edible fleshy leaves. Specifically, *C. cardunculus* L. shows three botanical varieties including the well-known globe artichoke variety (var. *scolymus* (L.)), cultivated or leafy cardoon (var. *altilis* DC.) and their wild perennial progenitor (var. *sylvestris* (Lamk)) [1]. The globe artichoke is widely investigated for its chemical profile and valued for its nutraceutical and medicinal properties [2,3,4,5,6,7]. Globe artichokes contain a very little amount of fats and high levels of minerals (potassium, sodium, phosphorus), vitamin C, fiber, inulin and polyphenols, hydroxycinnamates, and flavones [8,9]. The functional properties of the artichoke are related to its high levels of polyphenolic compounds and inulin [10,11,12]. In artichoke flower heads, the most abundant phenolic substances are caffeoylquinic acid derivatives, especially chlorogenic acid, cynarin, and flavonoids, mainly apigenin and luteolin, both present as glucosides and rutinosides [3]. The antioxidant capacity of the artichoke has been associated to its high phenolic content [2,13,14,15]. Phenolic profiles of different *C. cardunculus* genotypes show significant differences, but apigenin and caffeoylquinic acid derivatives were generally the main compounds in all the samples. Genotype influence has also been observed in relation to antioxidant activity [16,17,18]. Investigations performed on infusion, decoction, and hydroalcoholic extracts of artichoke leaves revealed different concentrations of phenolic compounds and differences in antioxidant activity [13,19,20]

The edible portion of the artichoke is also a rich source of inulin (19–32% on a dry matter basis), a carbohydrate reserve with a high value in human nutrition due to its prebiotic properties. Inulin resists digestion in the small intestine due to the β-(2→1) fructosyl-fructose linkages but can be fermented by colonic microflora, stimulating the proliferation of commensal bacteria such as *Bifidobacteria* spp. and *Lactobacillus* spp [3,21,22]. For these reasons, artichokes are a valuable source of prebiotic dietary fibers but also of low-calorie carbohydrates with the potential to be used in the production of fat-reduced foods. The positive effects consist in blood glucose attenuation, in the control of cholesterol and serum lipids levels, and in mineral bioavailability [21].

In Italy, the globe artichoke represents one of the most important horticultural crops, with a production of about 384,000 tons in 2021 [23]. Italy is also the richest source of artichoke germplasm, with numerous commercial and local varieties adapted to different environments, which can differ in chemical composition, especially of the polyphenolic fraction, hence exhibiting different nutraceutical and pharmacological properties [14]. “Carciofo di Paestum”, an Italian traditional late clone cultivar, which produces purple heads with light green coloration from March to May [8], is a labeled PGI (Protected Geographical Indication) product of the Campania region, representing an important economic resource.

Previous investigations aimed at assessing and quantifying polyphenols and hydroxycinnamic acids in the globe artichoke “Carciofo di Paestum” were performed mainly on methanol extracts by HPLC-DAD-ESI/MS^n^ [24,25]. A metabolomics approach to characterize CH_2_Cl_2_/MeOH/H_2_O extracts of “Carciofo di Paestum” heads was performed also by NMR analysis [26]. Moreover, “Carciofo di Paestum” by-products were investigated by UHPLC-UV-HRMS [21].

The term “nutraceuticals” derives from “nutrition” and “pharmaceuticals” and is used for nutrition products that are also used as medicine [27]. Nutraceuticals refer to compounds aimed at supplementing the normal diet, containing substances alone or in combination with vitamins and minerals, or herbal products. Thus, they are referred to as “dietary supplements,” or “food supplements” [27,28,29].

Their use has been steadily increasing all over the world for the last decades, in the form of capsules, liquids, pills, and tablets in measured doses. They are very attractive because of their relatively low prices, non-prescription status, huge promotion, and the perception that natural products are safe [29]. Food supplements are generally extracted with atoxic solvents, such as ethanol and water. Ethanol is the most common bio-solvent, completely biodegradable, and obtained by the fermentation of sugar-rich materials such as sugar beet and cereals; therefore, green solvents such as water and aqueous ethanol solutions are among the preferred ones for extraction processes [30]. Moreover, it is very common to drink the infusion (teas) made with artichoke leaves for liver and digestive issues [13]. In recent years, with an increasing interest in the development of green and environmentally friendly extraction methods, new technologies and methods of extraction have been used [31].

These considerations prompted us to investigate the chemical composition of ethanol: water and water extracts of the globe artichoke “Carciofo di Paestum” since only methanol extracts have thus far been analyzed. The extraction procedures can greatly affect the fingerprints of the extracts. To the best of our knowledge, this is the first comprehensive investigation of green extracts of “Carciofo di Paestum” by a combination of LC-MS and NMR analysis. Metabolomic analysis generates huge datasets that make the application of chemometric methods necessary. Multivariate data analysis (MVDA) was used to identify the metabolite variation among green extracts.

## 2. Results and Discussion

### 2.1. LC-ESI/QExactive/MS/MS Analysis of MeOH Extract of “Carciofo di Paestum” PGI

“Carciofo di Paestum” PGI heads were extracted with MeOH to compare this extract with those obtained by green extractions. To identify the specialized metabolites occurring in the MeOH extract of the artichoke, an analytical approach based on LC-MS was carried out [32].

Hyphenated techniques are playing increasingly important roles in support of phytochemical investigations, and in particular, high liquid chromatography (HPLC) coupled to mass spectrometry (MS) is considered a powerful tool [33]. In this case, high-performance liquid chromatography coupled with electrospray ionization and hybrid Quadrupole-Orbitrap mass spectrometry (LC-ESI/QExactive/MS/MS) was used.

**Figure 1:**
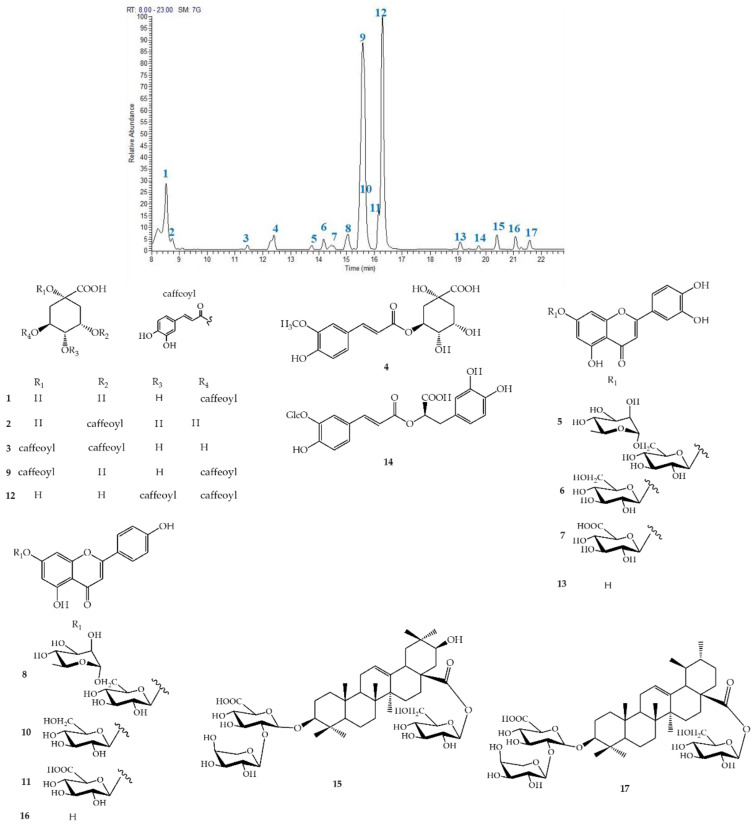
Specialized metabolites isolated from MeOH extract of *C. cardunculus* subsp. *scolymus*: cultivar “Carciofo di Paestum” PGI.

According to their accurate mass, characteristic fragmentation pattern, retention time, and literature data, 17 compounds (Figure 1), corresponding to caffeoylquinic acids (**1**, **2**), dicaffeoylquinic acids (**3**, **9**, **12**), phenolic compounds (**4**, **14**), flavonoid derivatives (**5**–**8**, **10**, **11**, **13**, **16**), and terpenoids (**15**, **17**), could be identified. In particular, on the basis of characteristic product ions at *m/z* 191 and 179, compounds **1** and **2** were identified as 5- and 3-caffeoylquinic acids, respectively [34]. The identity of 5-caffeoylquinic acid (chlorogenic acid) was confirmed by comparison with a standard compound. Dicaffeoylquinic acids were identified by their fragmentations at *m/z* 353, 335, and 191 [35,36]. Moreover, 1,5-dicaffeoylquinic acid (**9**) was confirmed by comparison with the reference compound. Regarding flavonoid derivatives, information on sugar moieties and aglycone was derived by characteristic fragmentation patterns. In particular, compounds **5**–**7** showed a characteristic fragment at *m/z* 285 corresponding to luteolin, whereas compounds **8**, **10**, and **11** displayed a typical product ion at *m/z* 269 corresponding to apigenin (Figure 1 and Table S1).

### 2.2. Isolation and Identification of Specialized Metabolites

To unambiguously identify specialized metabolites in artichoke extracts, isolation of compounds from MeOH extract by HPLC-UV was carried out. In this way, 17 compounds were isolated and characterized by NMR analysis. The isolated compounds were identified as: 5-caffeoylquinic acid (**1**), 3-caffeoylquinic acid (**2**), 1,3-dicaffeoylquinic acid (**3**), 5-feruloylquinic acid (**4**), luteolin-7-*O*-rutinoside (**5**), luteolin-7-*O*-β-D-glucopyranoside (**6**), luteolin-7-*O*-β-D-glucuronide (**7**), apigenin-7-*O*-rutinoside (**8**), 1,5-dicaffeoylquinic acid (**9**), apigenin-7-*O*-β-D-glucopyranoside (**10**), apigenin-7-*O*-β-D-glucuronide (**11**), 4,5-dicaffeoylquinic acid (**12**), luteolin (**13**), salviaflaside (**14**), cynarasaponin J (**15**), apigenin (**16**), and cynarasaponin A (**17**) (Figure 1).

### 2.3. Metabolite Fingerprint of “Carciofo di Paestum” PGI Green Extracts by LC-ESI/QExactive/MS/MS

To evaluate the possibility to use heads of *C. cardunculus* subsp. *scolymus* as a food supplement in the nutraceutical field but also in cosmetic formulations, simple and fast extraction methods based on the use of cheap and relatively non-toxic solvents were carried out [37]. EtOH was selected as a “green” solvent considering that it is a good solvent for polyphenol extraction and safe for human consumption. Therefore, the heads of “Carciofo di Paestum” PGI were extracted by maceration with EtOH and with different mixtures of EtOH:H_2_O (80:20, 70:30, 60:40); moreover, based on Pharmacopeia XII, the artichoke was submitted to infusion and decoction. All the obtained extracts were analyzed by LC-ESI/QExactive/MS/MS.

**Figure 2:**
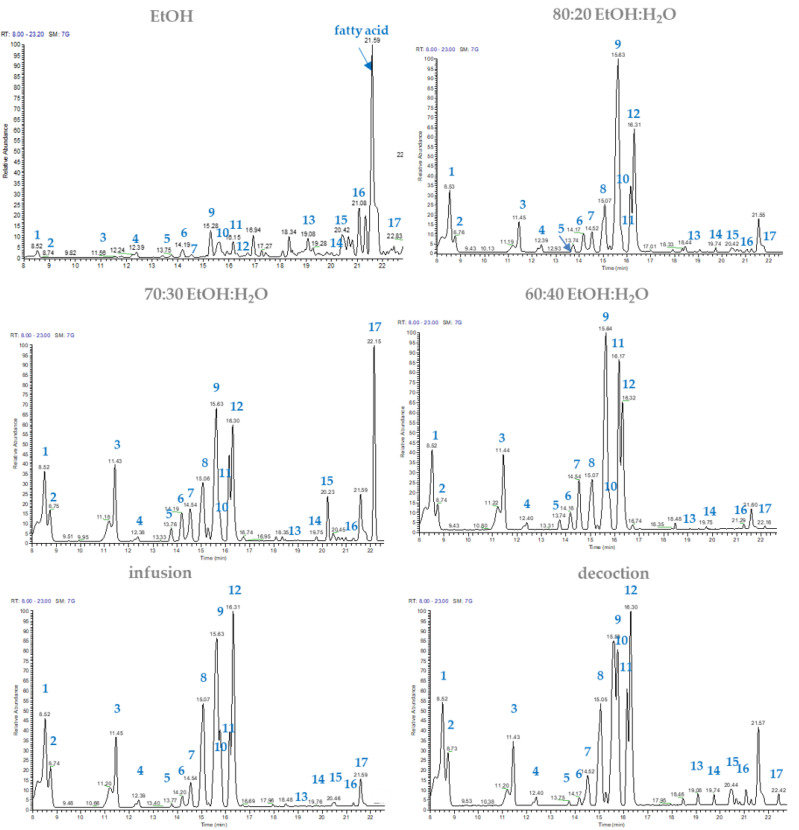
LC-ESI/QExactive/MS profile of different extracts of heads of “Carciofo di Paestum” PGI.

Among all the extracts, hydroalcoholic, infusion, and decoction extracts were the most interesting, showing higher peaks for flavonoids and quinic acid derivatives than MeOH extract; moreover, with respect to the latter, the “green” extracts allowed a better extraction of cynarasaponin J (**15**) and A (**17**) (Figure 2).

### 2.4. Targeted Multivariate Statistical Analysis of Secondary Metabolites

To identify similarities in the chemical profile among different samples, the metabolite profiles of different “green” extracts of the artichoke in triplicate were analyzed by multivariate statistical analysis. The raw data were first filtered using MZ mine 2.0 software and then processed using SIMCA-P+ software.

The result of the validation test further emphasized the significance and predictability of the model when the targeted approach was applied; in particular, PC1 contributed to 67.9% of the variance, followed by PC2, which contributed to 18.1%.

**Figure 3:**
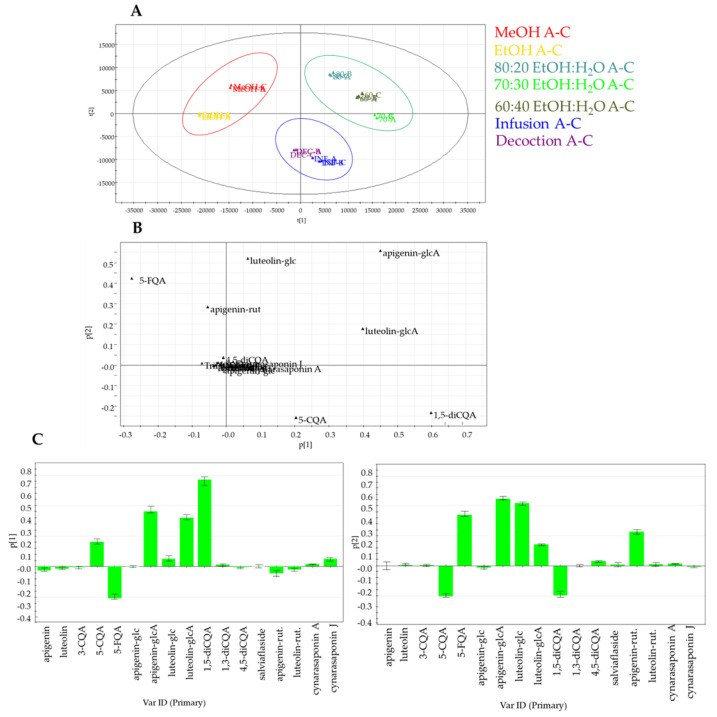
Principal component analysis of specialized metabolites in artichoke extracts obtained by LC-MS targeted analysis: (**A**) PCA score scatter plot; (**B**) PCA loading plot; (**C**) PCA loading column plots. CQA, caffeoylquinic acid; FQA, feruloylquinic acid; diCQA, dicaffeoylquinic acid; rut, rutinoside; glc, glucoside; glcA, glucuronide.

The Principal Component Analysis (PCA) highlighted significant differences among the extraction methods. Methanol extracts and the “eco-friendly” extracts were located on the opposite side of the plot, confirming the different selectivity of the employed methods. In the PCA score plot, three clusters could be considered, the first in the left region with EtOH and MeOH extracts, the second cluster in the upper right with hydroalcoholic extracts, and the third cluster in the lower region including infusion and decoction (Figure 3A). The PCA loading plot highlighted that hydroalcoholic extracts were richer than other extracts in flavonoid glycoside derivatives. Meanwhile, infusion and decoction were more abundant in 5-caffeoylquinic acid (5-CQA) and 1,5-dicaffeolylquinic acid (1,5-diCQA) (Figure 3B,C).

### 2.5. Quantitative Analysis of 5-Caffeoylquinic Acid (**1**) and 1,5-Dicaffeoylquinic Acid (**9**)

The two compounds 5-caffeoylquinic acid (chlorogenic acid) (**1**) and 1,5-dicaffeoylquinic acid (**9**) are reported as the main quinic acid derivatives occurring in *C. cardunculus* subsp. *scolymus* [21]. Thus, these two compounds were quantified in the different extracts of *C. cardunculus* subsp. *scolymus* heads by LC-ESI/QTrap/MS/MS analysis. Multiple Reaction Monitoring (MRM), a very accurate and sensitive tandem mass spectrometric technique, was used [38].

Based on the transitions selected for MRM experiments, the amount (mg/g of extract) of each compound in “green” and MeOH extracts was determined.

The compound 5-caffeoylquinic acid (**1**) occurred in a concentration range of 0.49–5.05 mg/g extract, showing the highest concentration in the infusion and decoction, according to PCA loading plot results; moreover, the abovementioned preparations were also the richest ones of 1,5-dicaffeoylquinic acid (**9**), although this compound was also very abundant in the other green preparations (Table 1 and Table S2).

### 2.6. Metabolite Fingerprint of Primary Metabolites of C. cardunculus subsp. scolymus “Green” Extracts by Multivariate Data Analysis based on ^1^H NMR

Food supplements are known to contain one or more dietary compounds such as vitamins, minerals, amino acids, carbohydrates, and other substances with a nutritional or physiological effect. As previously described, inulin represents a carbohydrate reserve with a high value in human nutrition, typically occurring in artichokes. To obtain complete information not only about the occurrence of specialized metabolites but also of primary metabolites, as inulin in the different extracts of “Carciofo di Paestum” PGI, a multivariate approach based on ^1^H NMR analysis has been carried out.

In the first step, to assign a key signal for each primary metabolite, ^1^H NMR of all extracts, in triplicate, were acquired; in this way, 12 primary metabolites including free amino acids, fatty acids, γ-aminobutyric acid, as well as sugars were identified. In detail, the following amino acids were assigned: isoleucine (0.94, t, J = 7.0 Hz), leucine (1.07, d, J = 7.0 Hz), alanine (1.47, d, J = 7.2 Hz), lysine (1.87, d, J = 8.0 Hz), glutamate (2.07, m), aspartic acid (2.75, dd, J = 16.0, 3.0 Hz), and phenylalanine (7.32, m); the key signals at 1.27 (s) and 2.27 (t, J = 7.2 Hz) were attributed to fatty acids and γ-aminobutyric acid (GABA), respectively. Moreover, in the carbohydrate region, ^1^NMR spectra displayed characteristic signals assigned to β-fructofuranoside (4.19, d, J = 2.3 Hz) and β-glucopyranoside (4.47, d, J = 8.0 Hz), as well as inulin (5.40, d, J = 5.0 Hz) (Figures S1–S8 and Table S3).

In the second step, to investigate the impact of the different solvents on the extraction of primary metabolites, PCA analysis was carried out. PCA was performed by measuring the selected peak area for each identified metabolite in the ^1^H-NMR dataset and by using these areas (variables) as rows of a matrix, while the different extracts of *C. cardunculus* subsp. *scolymus* (observations) were the columns of the matrix. The resulting model, obtained after scaling data by Pareto scaling, showed good fitness and the absence of outliers. PC1 contributed to 86.3% of the variance followed by PC2, which contributed to 7.7%. Thus, the first two PCs exhibited a total variance of 94.0%.

**Figure 4:**
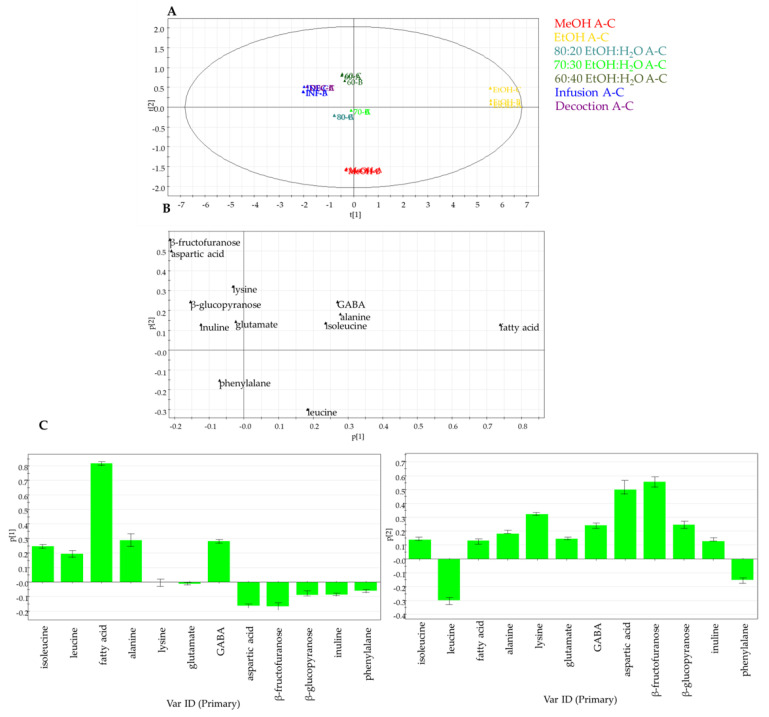
Principal component analysis of primary metabolites in artichoke extracts obtained by NMR targeted analysis: (**A**) PCA score scatter plot; (**B**) PCA loading plot; (**C**) PCA loading column plots.

A PCA score plot (Figure 4A) highlighted evident differences between EtOH and MeOH extracts with hydroalcoholic extracts and infusion and decoction. In detail, the PCA loading plot (Figure 4B,C) displayed how carbohydrates are characteristic of infusion and decoction, while fatty acids are typical of EtOH extract, in agreement with the polarity of the solvent used for extractions.

### 2.7. Evaluation of Phenolic Content and Antioxidant Activity of “Carciofo di Paestum” PGI Extracts

**Figure 5:**
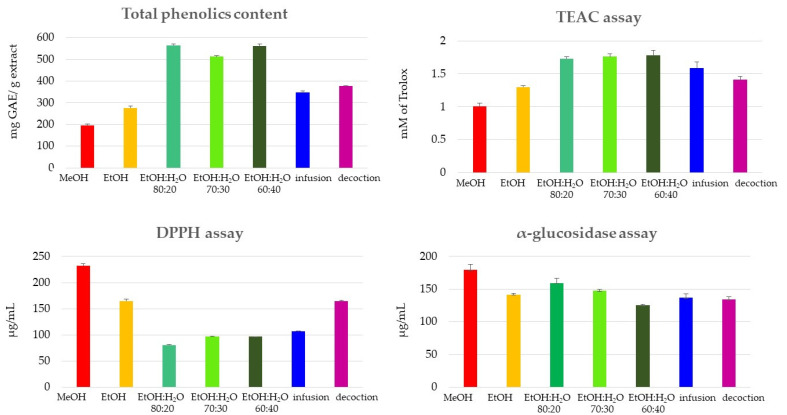
Phenolic content, TEAC, DPPH, and α-glucosidase assays of MeOH and green extracts of *C. cardunculus* subsp. *scolymus*, cultivar “Carciofo di Paestum” PGI.

ROS production has been demonstrated to contribute to the development of some diseases including cancer and cardiovascular diseases [39]. Polyphenols can react directly with ROS, preventing their concentrations from reaching harmful intracellular levels. To evaluate the radical scavenging activity by spectrophotometric assay, the preliminary phenolic content was tested by the Folin–Ciocalteu method [40]. The results of the total phenolic content determination indicated a high phenolic content for EtOH:H_2_O extracts. Mixtures EtOH:H_2_O 80:20, 70:30, and 60:40 showed GAE (milligrams of gallic acid equivalents for gram of extract) values corresponding to 565.14, 512.30, and 562.17, respectively. The high phenolic content shown by the EtOH:H_2_O extracts could be attributed to the synergistic effect of the two solvents in the extraction of phenolic compounds; in detail, water could have an important role in the swelling of plant material, whereas ethanol is responsible for disrupting the binding between the solutes and plant matrix (Figure 5 and Table S4).

Successively, the antioxidant activity of “Carciofo di Paestum” PGI extracts was tested by TEAC and DPPH assays. The TEAC assay highlighted a concentration-dependent free-radical scavenging activity for hydroalcoholic extracts as well as infusion and decoction. In detail, hydroalcoholic extracts displayed a radical scavenging activity (1.73–1.78 mM) comparable to quercetin (2.30 mM), used as a reference compound. The DPPH assay displayed how hydroalcoholic extracts exerted a promising antioxidant activity; in particular, EtOH:H_2_O 80:20 showed the strongest activity (EC_50_ = 80.51 µg/mL), followed by the other hydroalcoholic extracts and by infusion and decoction. The weakest antioxidant activities were shown by MeOH and EtOH extracts, confirming the results obtained by the TEAC assay (Figure 5 and Table S4).

The ABTS and DPPH values of different artichoke extracts against their corresponding total phenolic content (TPC) values were correlated using Pearson’s method (Table S5). Thaipong et al. reported that Pearson’s correlation coefficient was significantly negative if −0.61 ≤ r ≤ −0.97 and significantly positive if 0.61 ≤ r ≤ 0.97 [41]. In particular, there was a positive and significant correlation between TEAC and TPC. About the DPPH assay, the increase in TPC may be related to the increase in antioxidant activities, indicated by lower IC_50_ DPPH. Therefore, TPC was significantly negatively correlated with IC_50_ DPPH (Table S5).

### 2.8. α-Glucosidase Inhibitory Activities of “Carciofo di Paestum” PGI Extracts

The inhibitory effects of the “Carciofo di Paestum” PGI extracts against α-glucosidase were evaluated in comparison with those exerted by the antidiabetic compound acarbose. The enzyme α-Glucosidase is the key catalyzing enzyme involved in the process of carbohydrate digestion and glucose release. Inhibition of α-glucosidase is a very effective way of delaying glucose absorption and lowering the postprandial blood glucose level, which can potentially suppress the progression of diabetes mellitus [42].

Extracts and positive control (acarbose) were evaluated in the concentration range of 200–500 µg/mL. All tested extracts, except the MeOH extract, showed moderate activity with IC_50_ values ranging from 125.3 to 159.3 µg/mL, comparable to acarbose (Figure 5 and Table S6). In detail, EtOH:H_2_O 80:20 (IC_50_: 125.3) was more active than acarbose (IC_50_: 132.5), while the other hydroalcoholic extracts and the EtOH extract displayed an activity comparable to acarbose.

## 3. Materials and Methods

### 3.1. Chemicals

The solvent for extractions, LC-MS and NMR analysis, Folin-Ciocalteu phenol reagent, 2,2-Diphenyl-1-picrylhydrazyl (DPPH•), vitamin C, 2,2′-azino-bis(3-ethylbenzothiazoline-6-sulphonic acid) (ABTS), potassium persulfate (K_2_S_2_O_8_), phosphate-buffered saline (PBS) solution, Trolox, α-glucosidase enzyme (from Saccharomyces cerevisiae), and 4-nitrophenyl α-D-glucopyranoside (PNPG), were purchased from Sigma Aldrich (Milano, Italy).

### 3.2. Plant Material and Sample Preparation

The heads of *C. cardunculus* subsp.*scolymus*. cv. “Carciofo di Paestum” were collected at commercial maturity, at Paestum, Salerno, Italy in March 2017. A voucher specimen has been deposited in this department.

### 3.3. Sample Extraction

After harvest, the heads of *C. cardunculus* subsp. *scolymus*, cultivar “Carciofo di Paestum” were transported to the laboratory and crushed into little parts; immediately after, the heads were stored in the freezer at a temperature of −20 °C. After 10 days, they were submitted to lyophilization. At this point, a ratio of 2.0 g/200 mL solvent (*w*/*v*) of dried heads was used for each extraction, using as solvents MeOH, EtOH, EtOH: H_2_O (8:2), EtOH: H_2_O (7:3), EtOH: H_2_O (6:4), for 3 days, 3 times. After filtration and evaporation of the solvent to dryness in vacuo, dried extracts were obtained. In this way, four different “green” extracts, together with a MeOH extract, were obtained.

Based on Pharmacopeia XII, infusion and decoction were prepared.

The infusion was carried out by adding boiled distilled water to the plant with a specified solid–solvent ratio (1:10 *w*/*v*), leaving it in touch the solvent and the plant for 10 min under moderate stirring. After this time, stirring was stopped, and the solvent was filtrated to obtain the infusion that was submitted to the analysis.

For decoction, the plant was boiled in an open-type extractor in a specified solid–solvent ratio (1:20 *w*/*v*) for 2 h, using moderate stirring. Both beakers were covered by aluminum and protected with plastic parafilm both to permit the conservation of a stable temperature and to prevent solvent evaporation and light reaction; then the solvents were filtered under reduced pressure.

### 3.4. LC-ESI/QExactive/MS/MS Analysis

The extracts of *C. cardunculus* subsp.*scolymus* heads were analyzed using liquid chromatography coupled with electrospray ionization and a high-resolution mass spectrometer (QExactive: hybrid Quadrupole-Orbitrap Mass Spectrometer, ThermoFischer, Waltham, MA, USA), operating in negative ion mode.

LC-MS analysis was carried out on a Kinetex C18 2.6 mm (100 mm× 2.1 mm) column (Phenomenex, Aschaffenburg, Germany), using a flow rate of 0.2 mL/min. A binary solvent system was used (eluent A: water with 0.1% formic acid (99.9:0.1, *v*/*v*), eluent B: acetonitrile with 0.1% formic acid (99.9:0.1, *v*/*v*),). The HPLC gradient started at 5% B. After 10 min, % B was at 20%, after 20 min, it was at 60%, in 2 min % B arrived at 95%, holding at this percentage for 5 min, before returning to the starting percentage. The autosampler was set to inject 4 μL of each extract (1.0 mg/mL MeOH). The auxiliary gas was set at 10 (arbitrary units), and the sheath gas was set at 50 (arbitrary units).

### 3.5. Isolation of Specialized Metabolites

MeOH extract of *C. cardunculus* subsp. *scolymus* was analyzed by a RP-HPLC-UV system setting wavelength at 254 nm. The elution gradient was obtained using water with 0.1% formic acid as eluent A (99.9:0.1, *v*/*v*), and acetonitrile with 0.1% formic acid (99.9:0.1, *v*/*v*), as B at a flow rate of 2.0 mL/ min. A Phenomenex Sinergy Hydro Prep MS C18 column (250 mm × 10 mm, 10 micron) was used. The HPLC gradient started at 10% B and remained at 10% for 5 min; after 12 min, % B was at 20%, after 13 min, it was set at 26%, after 10 min, % B was at 38%, after 10 min, it was at 90%, and finally after 2 min at 100%; it was held at 100 % for 10 min before returning to the starting percentage. Compounds **1** (5.8 mg, *t*_R_ = 22.0 min), **2** (1.7 mg, *t*_R_ = 24.09 min), **3** (4.2 mg, *t*_R_ = 29.27 min), **4** (2.3 mg, *t*_R_ = 31.03 min), **5** (2.9 mg, *t*_R_ = 31.30 min), **6** (3.1 mg, *t*_R_ = 32.87 min), **7** (3.8 mg, *t*_R_ = 33.52 min), **8** (2.1 mg, *t*_R_ = 35.70 min), **9** (5.8 mg, *t*_R_ = 37.29 min), **10** (3.3 mg, *t*_R_ = 39.49 min), **11** (4.2 mg, *t*_R_ = 41.95 min), **12** (4.5 mg, *t*_R_ = 44.52 min), **13** (2.3 mg, *t*_R_ = 45.39 min), **14** (2.4 mg, *t*_R_ = 47.10 min), **15** (3.6 mg, *t*_R_ = 48.5 min), **16** (2.5 mg, *t*_R_ = 49.8 min), and **17** (4.6 mg, *t*_R_ = 50.6 min) were obtained.

### 3.6. Multivariate Data Analysis of Specialized Metabolites

Xcalibur 2.2 from Thermo Fisher Scientific was used to check the exact mass of each secondary metabolite identified by LC-MS analysis (negative ion mode). MZMine 2.10 was used for LC-MS data processing (http://mzmine.sourceforge.net (accessed on 10 March 2022)).

MZMine software was used to filter the noise and detect and align the peaks observed in LC-MS profiles (noise level 1.0 × 10^4^; all data points below this intensity level were ignored) after exporting the processed data in tabular format (.cvs file). Each sample was analyzed in triplicate.

For targeted analysis of specialized metabolites, the peak area obtained from LC/MS analysis was considered. Pareto scaling was applied before multivariate data analysis; in detail, the matrix was constituted of 17 variables, corresponding to the peak area of *m/z* values of specific specialized metabolites of *C. cardunculus* subsp. *scolymus*, and 7 observations, represented by different extracts.

### 3.7. Quantitative Analysis of 5-Caffeoylquinic Acid (**1**) and 1,5-dicaffeoylquinic Acid (**9**)

Quantitative analyses were performed on a LC-ESI/QTrap/MS system working in Multiple Reaction Monitoring (MRM) mode. HPLC separation was conducted by Kinetex Omega μm RP C18 column (100 mm × 2.1 mm i.d) at a flow rate of 0.3 μL/min.

Linear gradient elution was carried out by using H_2_O with 0.1% formic acid as eluent A (99.9:0.1, *v*/*v*), and acetonitrile with 0.1% formic acid (99.9:0.1, *v*/*v*), as eluent B. The HPLC gradient started at 5% B; after 2.1 min, % B was at 15%, changing from 15% B to 35% B in 4.3 min, from 35% B to 80% B in 2.1 min, and returning to the starting percentage in 2.1 min. The instrument operated in the negative ion mode for internal standard (resveratrol, 1µg/mL for each solution) and external standards 5-caffeoylquinic acid (**1**) and 1,5-dicaffeoylquinic acid (**9**) specific values of declustering potential, focusing potential, entrance potential, collision energy, and collision cell exit potential were used (Table S2). *C. cardunculus* subsp. *scolymus* extracts were diluted by using methanol, and 5 μL (1.0 mg/mL) were injected in triplicate; solutions of different ES concentrations (0.5, 1.0, 5.0, 10, 20, 30, 50, and 70 µg/mL) were used. Linear regression analysis was performed using the Analyst 1.6.2 Software provided by the manufacturer (AB Sciex, Milan, Italy).

Linearity was evaluated by correlation values of calibration curves. The limit of quantification (LOQ; equivalent to sensitivity) was evaluated by injecting a series of increasingly diluted standard solutions until the signal-to-noise ratio was reduced to 10. The limit of detection (LOD) was estimated by injecting a series of increasingly diluted standard solutions until the signal-to-noise ratio was reduced to 3 [43] (Table S2).

### 3.8. NMR Analysis and Data Processing

NMR analyses were carried out on a Bruker Ascend-600 spectrometer (Bruker BioSpin GmBH, Rheinstetten, Germany) equipped with a Bruker 5 mm. Methanol-*d*_4_ (99.95%, Sigma-Aldrich) was used as solvent for each extract. The NMR data were processed using TopSpin 3.2 software. For HSQC, a spectral width of 12 ppm and 165 ppm in the proton and carbon dimensions, respectively, were used with 1 K data points, 64 scans, 256 t1 increments, and a recycle delay of 2 s. HMBC was obtained with a spectral width of 12 ppm and 230 ppm in the proton and carbon dimensions, respectively, 4 K data points, 120 scans, 256 t1 increments, and a recycle delay of 2 s.

For the targeted multivariate analysis, all samples were acquired in methanol-*d*_4_ (99.95%, Sigma-Aldrich). The peak of TPS (3-(Trimethylsilyl)-propionic-2,2,3,3-d_4_ acid sodium salt at 0.9% (*w*/*w*) in D_2_O 99.9 atom%) at 0 ppm was used as the chemical shift external reference. All samples were run at 300 K, using the zgesgp pulse sequence; the relaxation delay was 4.0 s, and the acquisition time was 5.45 s, with 128 number scans and data collected into 64 k data points. Each free induction decay (FID) was zero-filled to 128 k data points. Before Fourier transformation, an exponential window function with a line broadening factor of 0.3 Hz was applied. After the acquisition, the spectra were analyzed by MestreNova 10 software. In particular, the spectra were manually phased and the baseline corrected. Spectra were referenced using the TSP, obtaining good peak alignment. Bucketing was performed within the 0.5–8.2 ppm region (spectral buckets of 0.004 ppm), excluding the signals of the residual non-deuterated methanol and deuterated methanol. The obtained dataset was normalized by range area (0.5–8.2 ppm) normalization. Finally, the spectra were converted to an ASCII format. Each sample was analyzed in triplicate [37].

Pareto scaling was applied before multivariate data analysis; in detail, the matrix was constituted of 12 variables, corresponding to key chemical shift of primary metabolites of *C. scolymus*, and 7 observations, represented by different extracts.

### 3.9. Total Phenolic Content, DDPH, and TEAC Assays

Folin–Ciocalteu, DPPH, and TEAC assays for each extract have been performed as previously reported with slight modification [44].

The total phenolic content of the extracts was determined by Folin–Ciocalteu assay; gallic acid was used as a reference compound (calibration equation: y= 0.0038x + 0.145, R^2^ = 0.998). All the experiments were performed in triplicate, and results were expressed as the means of gallic acid equivalents (GAEmg/g dried extract) (Table S4).

Radical scavenging activity was determined by spectrophotometric assays of DPPH and TEAC. In particular, in the TEAC assay, the antioxidant activities of analyzed extracts (range= 0.25–1.00 mg/mL) were expressed as TEAC values in comparison with the TEAC activity of quercetin; TEAC values are expressed as concentration (mM) of a standard Trolox solution exerting the same antioxidant activity of a 1 mg/mL solution of the tested extract (calibration equation for Trolox: y= 31.683x + 52.436, R^2^ = 1.00) (Table S4).

For DPPH, the percentage of DPPH^•^ radical scavenging activity (%) was plotted against the extract concentration (µg/mL) to determine the IC_50_. In brief, stock solutions (1 mg/mL) of all the obtained extracts were used in the range of 50–200ug/mL, and an aliquot (5.0 µL) of the methanol solution containing different amounts of each extract was added to 195 µL of daily prepared DPPH^•^ solution. Absorbance at 517 nm was measured on a UV-visible spectrophotometer (Multiskans skyhigh, Thermo Fisher Scientific, Milan, Italy) 10 min after starting the reaction.

All the experiments were performed in triplicate. Range = 50–200 µg/mL was tested for each extract, and vitamin C was used as positive control and analyzed by linear regression [45] (Table S4).

### 3.10. α-Glucosidase Inhibition Assay

All α-Glucosidase inhibitory activity was evaluated as previously reported in Masullo 2022 [36,42]. In detail, 0.1 M phosphate buffer at pH 7.0 (150 μL) was added to each well; after 10 μL of samples (dissolved in MeOH at concentrations of 200, 300, 500 μΜ) were added, the reaction was started by the addition of 15 μL of the α-glucosidase enzyme (2 U/mL) in each well, and the plate was incubated for 5 min at 37 °C; after 5 min, 75 μL of the substrate (2.5 mM) 4- nitrophenyl α-D-glucopyranoside were added, and the plate was submitted to incubation for 10 min at 37 °C. The absorbance was measured at 405 nm before (T0′) and after the incubation with the enzyme (T10′). Acarbose was used as positive control, and negative control absorbance (phosphate buffer in place of the sample) was also evaluated. Inhibition of the enzyme was calculated, and the results were expressed as IC_50_ values. All experiments were carried out in triplicate.

IC_50_ values were calculated through non-linear regression using Graphpad Prism 5 Software and expressed as means of the standard deviation (SD) of three independent experiments. The results were considered statistically significant with values of *p* < 0.05.

### 3.11. Statistics and Data Analysis

Values were expressed as the average values ± standard deviation (SD) of at least triplicate experiments. The correlation coefficients among means were determined using Pearson’s method (Table S5) [41].

## 4. Conclusions

Considering the increasing interest in the production of artichoke-based food supplements, this work was aimed at defining the chemical profile of the green extracts of “Carciofo di Paestum” PGI. For the first time, a comprehensive investigation of green extracts was performed by LC-MS and NMR analysis to highlight the occurrence of both specialized and primary metabolites, respectively. Moreover, the use of statistical analysis to process the obtained data provided easy and clear visualization of the best extraction method. The globe artichoke is a source of caffeoylquinic acids, responsible for its healthy properties. 

Among the occurring caffeoylquinic acids, 5-caffeoylquinic acid known as chlorogenic acid represents one of the most available acids among phenolic acid compounds [46]. In addition, 5-caffeoylquinic acid and 1,5-dicaffeoylquinic acid known as cynarin play several important and therapeutic roles such as antioxidant activity, antibacterial, anticancer, hepatoprotective, cardioprotective, anti-inflammatory, antipyretic, neuroprotective, antiviral, anti-microbial, anti-hypertensive, free radicals scavenging, and central nervous system stimulating [46,47]. Literature data showed how 5-caffeoylquinic acid is able to modulate lipid and glucose metabolisms in metabolic-related disorders [46]. Recent reports have highlighted the role of 1,5-dicaffeoylquinic acid in the treatment of Alzheimer’s disease [47]. Considering the interesting activity reported for these molecules, this work highlights how 5-caffeoylquinic acid (5-CQA) and 1,5-dicaffeolylquinic acid (1,5-diCQA), occur in the highest amount in infusion and decoction. Noteworthy, the mixtures ethanol:water exerted a better ability to extract flavonoid glycosides.

The radical scavenging activity of the extracts is predominantly ascribed to phenolic compounds. According to the results obtained by LC-MS multivariate data analysis, the ethanol–water extracts showed the best total phenolic content as well as the best radical scavenging activity measured by DPPH assay.

A comprehensive chemical profile of the extracts is necessary to highlight the occurrence of primary metabolites such as amino acids and carbohydrates. This approach allowed us to identify the occurrence in *C. cardunculus* subsp. *scolymus* of inulin which represents one of the molecules responsible for artichoke health benefits. Preparations as infusions and decoctions contain a higher amount of inulin than methanol and ethanol extracts.

In conclusion, this work highlights the differences in the chemical profile of extracts obtained with different solvents and is aimed at providing clear and punctual knowledge on the chemical composition of Carciofo di Paestum PGI for its potential use as a food supplement.

## Figures and Tables

**Table 1:** Quantitative results of compounds **1** and **9** (mg/g extract ± SD) in extracts of *C. cardunculus* subsp. *scolymus* heads.

Compound	MeOH	EtOH	EtOH:H_2_O 80:20	EtOH:H_2_O 70:30	EtOH:H_2_O 60:40	Infusion	Decoction
5-caffeoylquinic acid (**1**)	0.49 ± 0.05	2.15 ± 0.30	1.71 ± 0.12	1.36 ± 0.20	0.90 ± 0.04	5.05 ± 0.71	2.90 ± 0.13
1,5-dicaffeoylquinic acid (**9**)	26.15 ± 3.88	27.85 ± 0.41	58.05 ± 0.92	30.45 ± 0.50	36.60 ± 1.27	87.65 ± 6.29	65.50 ± 4.50

## Data Availability

The data presented in this study are available in supplementary materials.

## Supplementary Materials

The following supporting information can be downloaded at: https://www.mdpi.com/article/10.3390/molecules27103328/s1, Table S1. Compounds identified in *C. scolymus* MeOH extract by LC-ESI/QExactive/MS/MS (negative ion mode); Table S2. LC–MS/MS conditions for quantitation of compounds **1** and **9** by negative ion MRM mode; Figures S1–S7. ^1^H NMR Spectra (600 MHz, CD_3_OD) of extracts; Figure S8. ^1^H NMR Spectrum with annotations of identified primary metabolites detected in *C. scolymus* heads EtOH H_2_O (80:20) extract; Table S3. Characteristic ^1^H NMR peaks of primary metabolites identified in *C. scolymus* extracts. Table S4. Phenolic content and antioxidant activity of green extracts of “Carciofo di Paestum” PGI extracts.; Table S5. Correlation between TPC evaluated by Folin–Ciocalteu and antioxidant activity evaluated by the ABTS, and DPPH methods. The correlation coefficients among means were determined using Pearson’s method; Table S6. α-glucosidase inhibitory activity of “Carciofo di Paestum” PGI extracts.

## Sample Availability

Samples of the compounds **1**–**17** are available from the authors.
