# Supplementary material for: Metabolite Profiling of “Green” Extracts of Cynara cardunculus subsp. scolymus, Cultivar “Carciofo di Paestum” PGI by 1H NMR and HRMS-Based Metabolomics
Source: Molecules. 2022 May 22;27(10):3328. doi: 10.3390/molecules27103328 (PMC9145539; doi:10.3390/molecules27103328)
Supplement: Supplementary file 1 [file molecules-27-03328-s001.zip › molecules-1724314-supplementary.pdf]

Supplementary material

Table S1. Compounds identified in *C. scolymus* MeOH extract by LC-ESI/Q-Exactive /MS/MS (negative ion mode).

Table S2. LC–MS/MS conditions for quantitation of compounds **1** and **9** by negative ion MRM mode.

Figure S1. ^{1}H NMR Spectrum (600 MHz, CD_3OD) of MeOH extract

Figure S2. ^{1}H NMR Spectrum (600 MHz, CD_3OD) of EtOH extract

Figure S3. ^{1}H NMR Spectrum (600 MHz, CD_3OD) of EtOH:H_2O 80:20

Figure S4. ^{1}H NMR Spectrum (600 MHz, CD_3OD) of EtOH:H_2O 70:30

Figure S5. ^{1}H NMR Spectrum (600 MHz, CD_3OD) of EtOH:H_2O 60:40

Figure S6. ^{1}H NMR Spectrum (600 MHz, CD_3OD) of infusion

Figure S7. ^{1}H NMR Spectrum (600 MHz, CD_3OD) of decoction

Figure S8. ^{1}H NMR Spectrum with annotations of identified primary metabolites detected in *C. scolymus* heads EtOH H_2O (80:20) extract.

Table S3. Characteristic ^{1}H NMR peaks of primary metabolites identified in *C. scolymus* extracts.

Table S4. Phenolic content and antioxidant activity of green extracts of "Carciofo di Paestum"

Table S5. Correlation between TPC evaluated by Folin-Ciocalteu and antioxidant activity evaluated by the ABTS, and DPPH methods. The correlation coefficients among means were determined using Pearson's method.

Table S6. α glucosidase inhibitory activity of "Carciofo di Paestum" PGI extracts.

Table S1. Compounds identified in *C. scolymus* MeOH extract by LC-ESI/Q-Exactive /MS/MS (negative ion mode).

	Compound	R_t (min)	Molecular Formula	$[M-H]^-$	Δ ppm	Characteristic product ions
**1**	5-caffeoylquinic acid (chlorogenic acid)	8.54	$C_{16}H_{18}O_9$	353.0876	2.38	191 (100), 179 (5), 135 (1)
**2**	3-caffeoylquinic acid (neochlorogenic acid)	8.77	$C_{16}H_{18}O_9$	353.0877	2.83	179 (74), 135 (27)
**3**	1,3-dicaffeoylquinic acid	11.46	$C_{25}H_{24}O_{12}$	515.1196	2.27	191 (40), 179 (100), 135 (49)
**4**	5-feruloylquinic acid	12.39	$C_{17}H_{20}O_9$	367.1026	0.60	173
**5**	luteolin-7-*O*-rutinoside	13.75	$C_{27}H_{30}O_{15}$	593.1495	0.97	447, 285
**6**	luteolin-7-*O*-β-D-glucopyranoside	14.17	$C_{21}H_{20}O_{11}$	447.0931	2.13	285
**7**	luteolin-7-*O*-β-D-glucoronide	14.47	$C_{21}H_{18}O_{12}$	461.0728	3.09	285
**8**	apigenin-7-*O*-rutinoside	15.05	$C_{27}H_{30}O_{14}$	577.1549	0.49	269
**9**	1,5-dicaffeoylquinic acid (cynarin)	15.58	$C_{25}H_{24}O_{12}$	515.1187	0.48	353, 191 (1), 179 (16)
**10**	apigenin-7-*O*-β-D-glucopyranoside	15.75	$C_{21}H_{20}O_{10}$	431.0972	-0.06	269
**11**	apigenin-7-*O*-β-D-glucuronide	16.16	$C_{21}H_{18}O_{11}$	445.0763	-0.20	269
**12**	4,5-dicaffeoylquinic acid	16.29	$C_{25}H_{24}O_{12}$	515.1186	0.36	353, 335, 191 (1), 179 (16)
**13**	luteolin	19.08	$C_{15}H_{10}O_6$	285.0405	4.09	151, 133
**14**	salviaflaside	19.74	$C_{24}H_{26}O_{13}$	521.1304	2.69	313, 298
**15**	cynarasaponin J	20.22	$C_{47}H_{74}O_{19}$	941.4747	0.72	779, 629
**16**	apigenin	21.07	$C_{15}H_{10}O_5$	269.0454	3.68	225, 117
**17**	cynarasaponin A	21.80	$C_{47}H_{74}O_{18}$	925.4792	0.05	763, 613

Table S2. LC–MS/MS conditions for quantitation of compounds **1** and **9** by negative ion MRM mode.

Compound	MRM transition	R^2	Regression line	DP	CE	EP	CXP	LOD	LOQ
5-caffeoylquinic acid (**1**)	353 -> 191	0.99	y= 0.00319x + 3.31	-60.0	-24.0	-4.0	-17.0	0.02	0.07
1,5-dicaffeoylquinic acid (**9**)	515 -> 353	0.99	y= 6.77e-6x + 0.38	-61.0	-24.0	-4.0	-38.0	0.25	0.84
resveratrol (internal standard)	227 -> 143	-	-	-37.0	-32.0	-10.0	-18.0	-	-

DP, Declustering Potential; **CE**, Collision energy; **EP**, Entrance potential; **CXP**, Collision Cell Exit Potential; LOQ, limit of quantification; LOD, limit of detection; LOD and LOQ expressed as μg/μL.

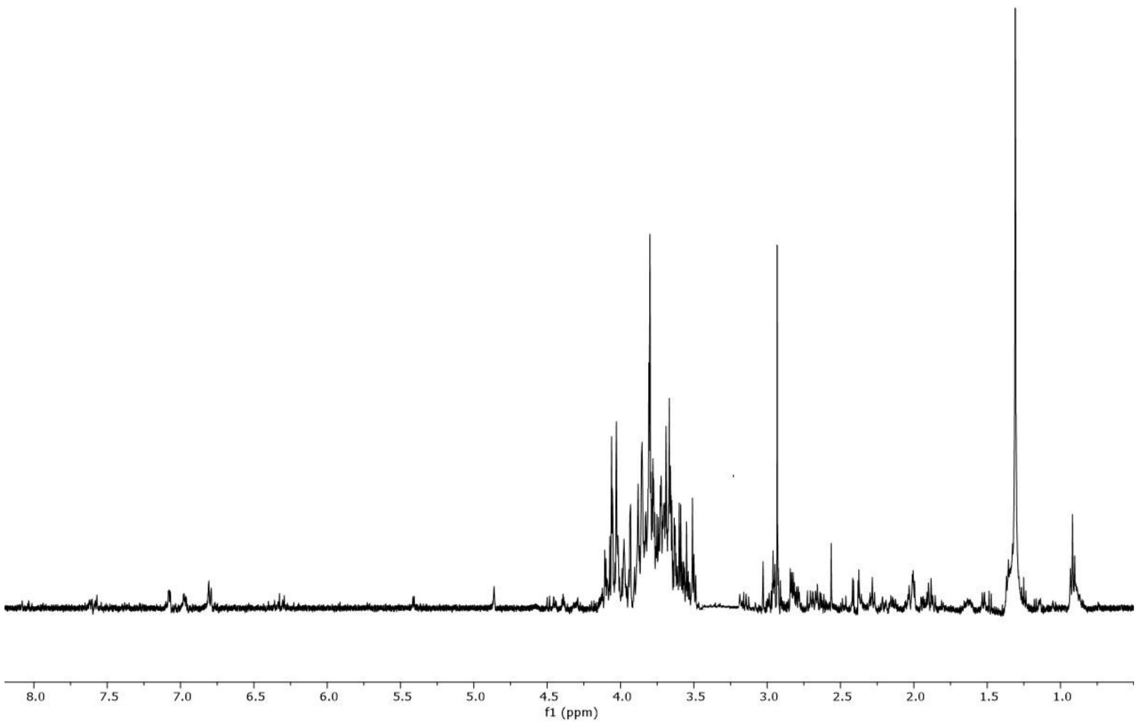

Figure S1. ^{1}H NMR Spectrum (600 MHz, CD_3OD) of MeOH extract

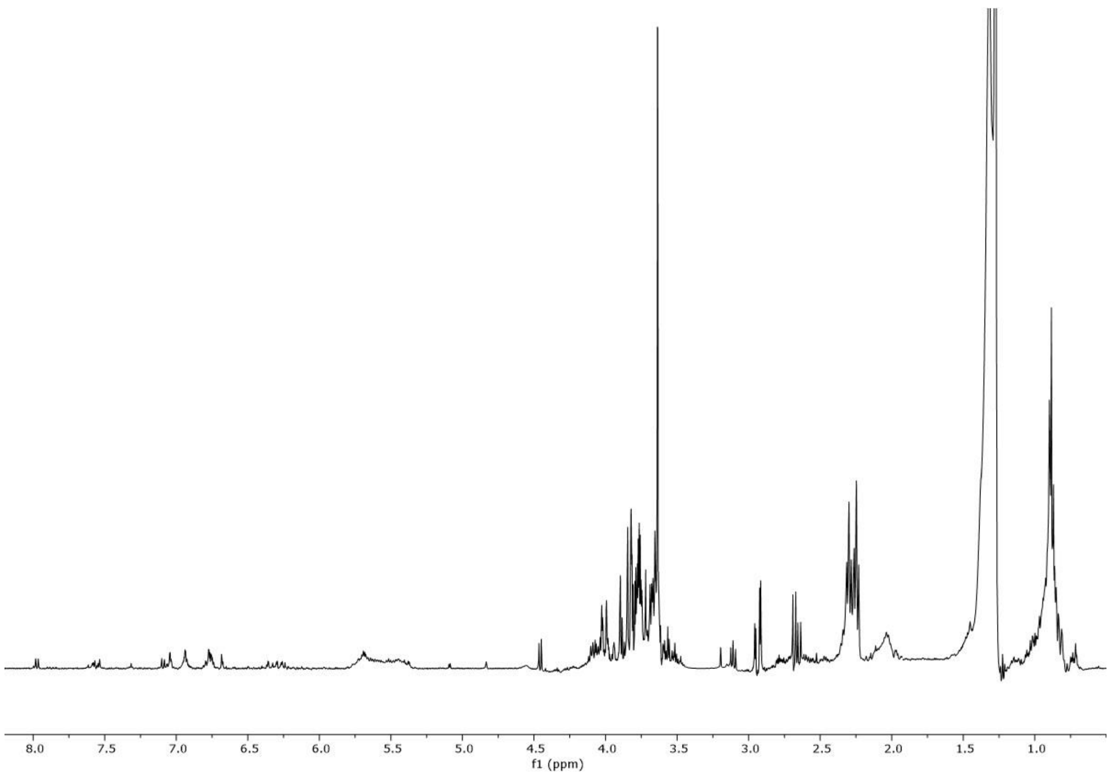

Figure S2. ^{1}H NMR Spectrum (600 MHz, CD_3OD) of EtOH extract

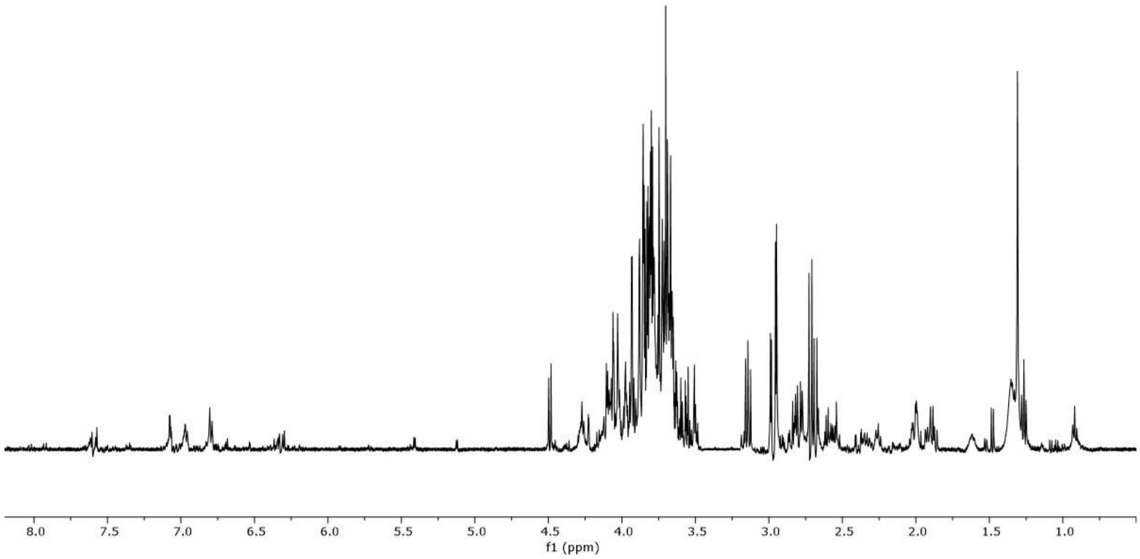

Figure S3. ^{1}H NMR Spectrum (600 MHz, CD_3OD) of EtOH:H_2O 80:20

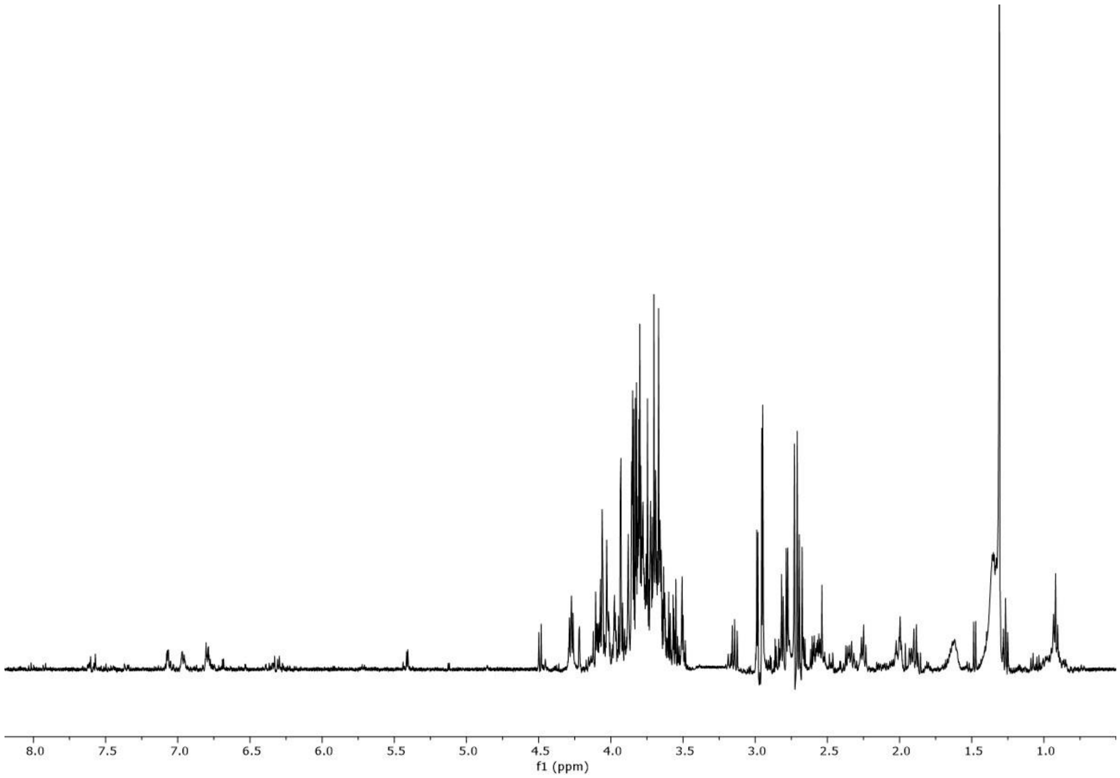

Figure S4. ^{1}H NMR Spectrum (600 MHz, CD_3OD) of EtOH:H_2O 70:30

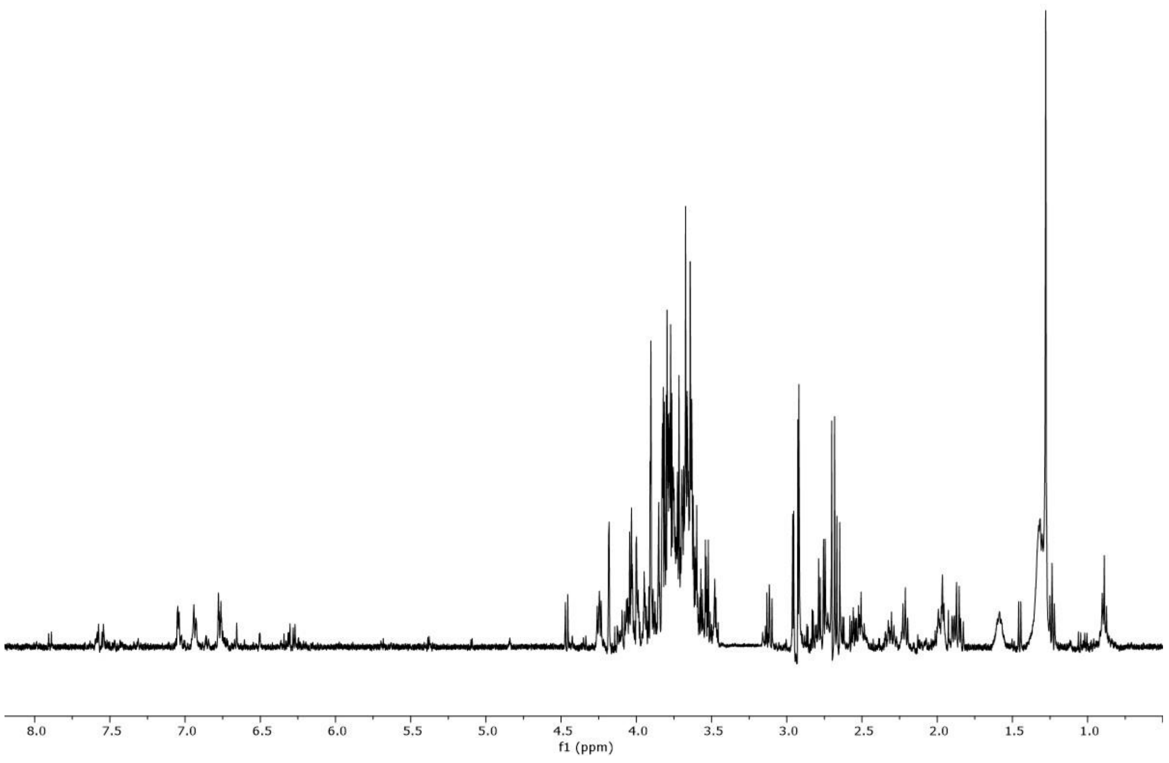

Figure S5. ^{1}H NMR Spectrum (600 MHz, CD_3OD) of EtOH:H_2O 60:40

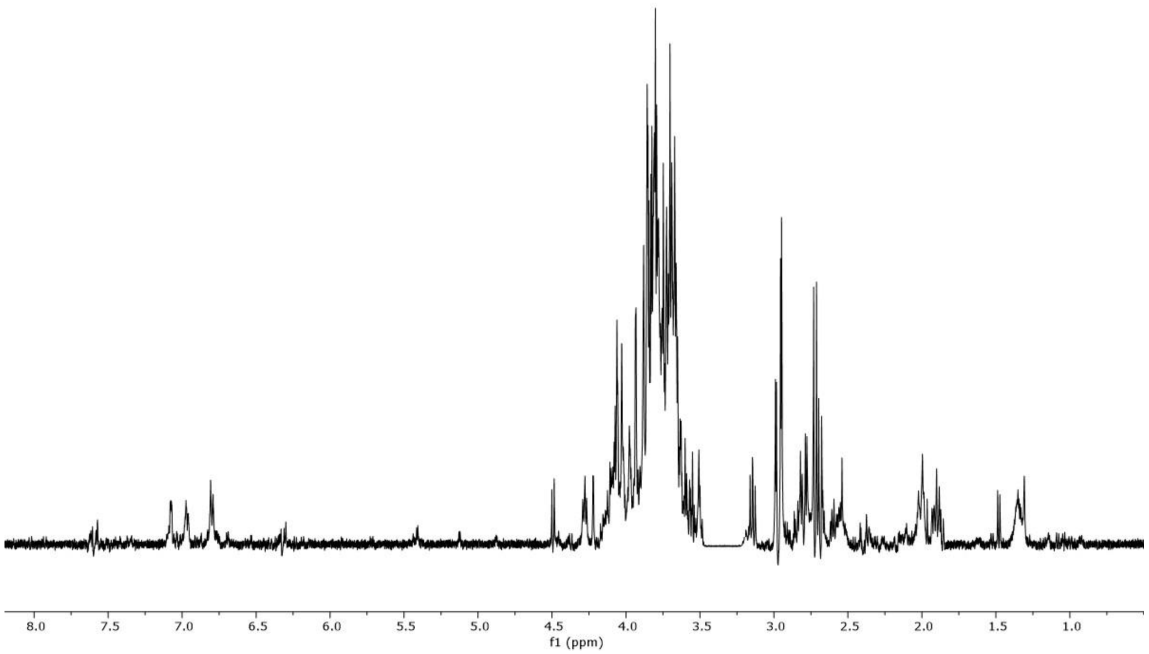

Figure S6. ^{1}H NMR Spectrum (600 MHz, CD_3OD) of infusion

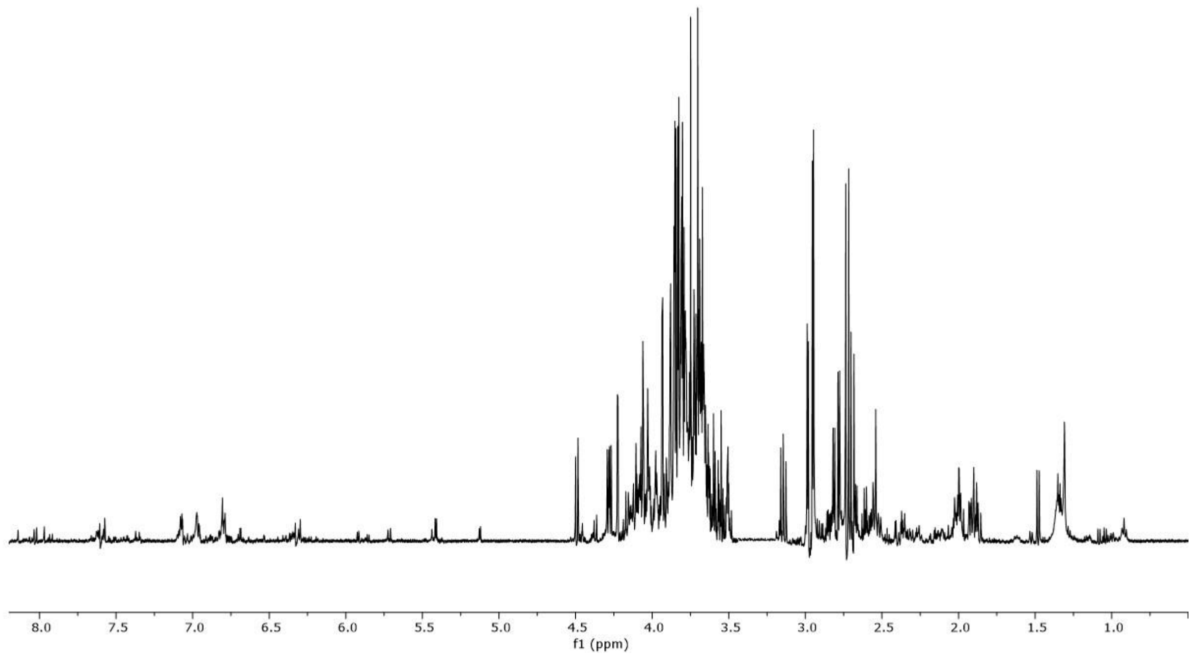

Figure S7. ^{1}H NMR Spectrum (600 MHz, CD_3OD) of decoction

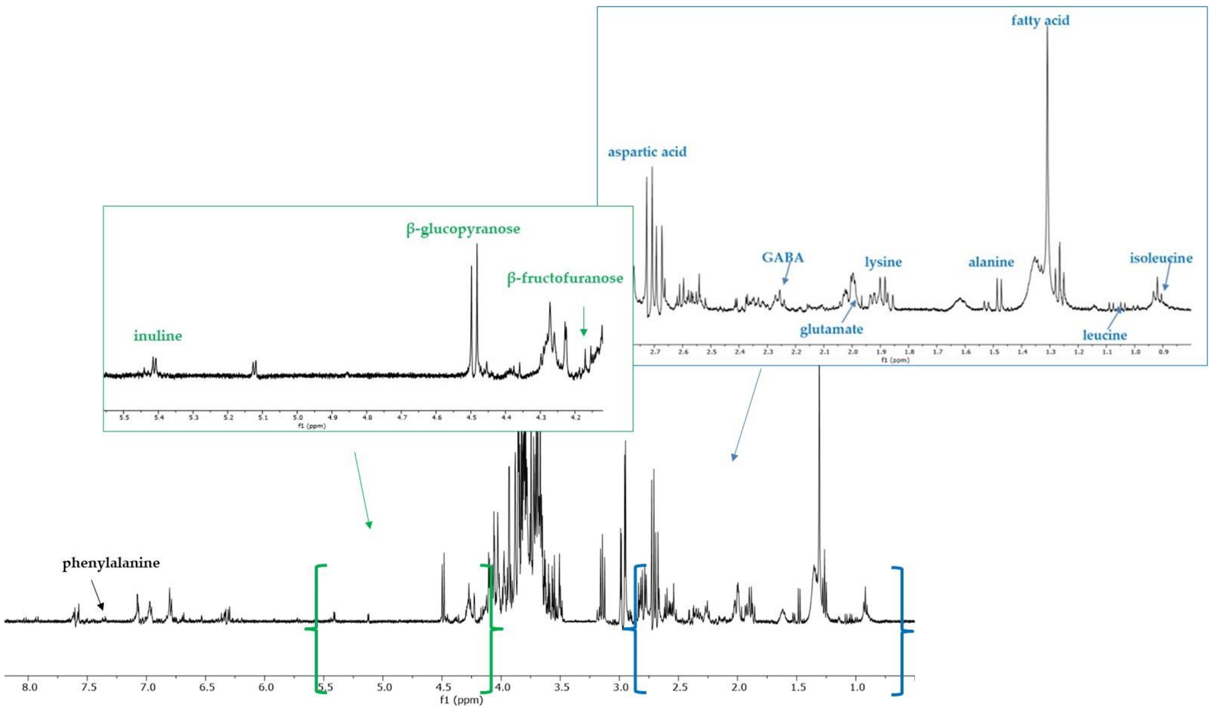

Figure S8. ^{1}H NMR Spectrum with annotations of identified primary metabolites detected in *C. scolymus* heads EtOH H_2O (80:20) extract.

Table S3. Characteristic ^{1}H NMR peaks of primary metabolites identified in *C. scolymus* extracts.

compound	^{1}H chemical shift (multiplicity, *J* in Hz)
isoleucine	0.94 (t, 7.0)
leucine	1.07 (d, 7.0)
fatty acid	1.27 (s)
alanine	1.47 (d, 7.2)
lysine	1.87 (d, 8.0)
glutamate	2.07 (m)
GABA	2.27 (t, 7.2)
aspartic acid	2.75 (dd, 3.0, 16.0)
β-fructofuranose	4.19 (d, 2.3)
β-glucopyranose	4.47 (d, 8.0)
inuline	5.40 (d, 5.0)
phenylalanine	7.32 (m)

Table S4. Phenolic content and antioxidant activity of green extracts of "Carciofo di Paestum"

	**Total phenolics content**	**DPPH$^\cdot$**	**ABTS$^{\cdot+}$**
***C.scolymus* extract**	**GAE[a] ± SD[b]**	**IC_{50}[c]± SD[b]**	**TEAC[d]± SD[b]**
MeOH	195.25 ± 6.65	231.71 ± 4.9	1.01 ± 0.05
EtOH	276.25 ± 8.89	165.02 ± 3.02	1.30 ± 0.02
EtOH:H_2O 80:20	565.14 ± 6.00	80.51 ± 1.00	1.73 ± 0.03
EtOH:H_2O 70:30	512.30 ± 4.44	97.30 ± 0.31	1.76 ± 0.04
EtOH:H_2O 60:40	562.17 ± 9.63	96.53 ± 0.12	1.78 ± 0.08
Infusion	347.41 ± 7.41	106.42 ± 0.40	1.59 ± 0.09
decotion	377.48 ± 1.48	164.96 ± 0.65	1.41 ± 0.05
Vitamin C[e]	14.93 ± 0.10		
Quercetin[f]			2.30 ± 0.08

[a] Values are expressed as milligrams of gallic acid equivalents (GAE) per gram of dried extract (mg GAE/g dried extract).; [b] SD: Results are expressed as mean of three experiments; SD, standard deviation. [c] Values are expressed as micrograms per milliliter (μg/mL), concentrations of extracts 50-200ug/ml [d] Values are expressed as concentration (mM) of a standard Trolox solution exerting the same antioxidant activity of a 1 mg/mL solution of the tested extract, concetration of extracts 0.25-1.0 mg/ml. [e]standard compound for DPPH assay, concentrations used 50-200 ug/ml. [f]standard compound for TEAC assay, concentrations used 0.3-1.5 mM.

Table S5. Correlation between TPC evaluated by Folin-Ciocalteu and antioxidant activity evaluated by the ABTS, and DPPH methods. The correlation coefficients among means were determined using Pearson's method.

Assay	Artichoke extracts R^2
TEAC	0.94
DPPH	-0.89

Table S6. α-glucosidase inhibitory activity of "Carciofo di Paestum" PGI extracts.

Artichoke extracts	IC_{50} ± SD (μg/mL)

MeOH	179.4± 8.32
EtOH	141.6 ± 2.35
EtOH:H_2O 60:40	159.3 ± 7.10
EtOH:H_2O 70:30	147.3 ± 2.84
EtOH:H_2O 80:20	125.3 ± 1.86
infusion	137.1 ± 5.28
decoction	134.4± 3.93
acarbose	132.5 ± 2.90
